# Effects of carbohydrate and caffeine mouth rinsing on strength, muscular endurance and cognitive performance

**DOI:** 10.1186/s12970-021-00462-0

**Published:** 2021-09-26

**Authors:** Raci Karayigit, Ajmol Ali, Sajjad Rezaei, Gulfem Ersoz, Angel Lago-Rodriguez, Raúl Domínguez, Alireza Naderi

**Affiliations:** 1 Faculty of Sport Sciences, Ankara University, Ankara, Turkey; 2 School of Sport, Exercise and Nutrition, Massey University, Auckland, 0745 New Zealand; 3 Physical Education and Sport Sciences Department, Faculty of Humanities, Tarbiat Modares University, Tehran, Iran; 4 Faculty of Health Sciences, Universidad Isabel I, 09004 Burgos, Spain; 5 Departmento de Motricidad Humana y Rendimiento Deportivo, Universidad de Sevilla, 41013 Sevilla, Spain; 6 Department of Exercise Physiology, Borujerd Branch, Islamic Azad University, Borujerd, Iran

**Keywords:** Ergogenic aid, Female athletes, Resistance exercise, Mouth rinse

## Abstract

**Background:**

Carbohydrate (CHO) and caffeine (CAF) mouth rinsing have been shown to enhance endurance and sprint performance. However, the effects of CHO and CAF mouth rinsing on muscular and cognitive performance in comparison between male and female athletes are less well-established. The aim of this study was to examine the effect of CHO and CAF rinsing on squat and bench press 1 repetition maximum (1-RM) strength, 3 sets of 40% of 1-RM muscular endurance and cognitive performance in both male and female athletes.

**Methods:**

Thirteen male and fourteen female resistance-trained participants completed four testing sessions following the rinsing of 25 ml of i) 6% of CHO (1.5 g); ii) 2% CAF (500 mg), iii) combined CHO and CAF (CHOCAF) solutions or iv) water (PLA) for 10 s. Heart rate (HR), felt arousal (FA), ratings of perceived exertion (RPE) and glucose (GLU) were recorded throughout the test protocol.

**Results:**

There were no significant differences in squat and bench press 1-RM, HR, RPE and GLU (*p* > 0.05) for males and females, respectively. FA was significantly increased with CAF (*p* = 0.04, *p* = 0.01) and CHOCAF (*p* = 0.03, *p* = 0.01) condition in both males and females, respectively. Squat endurance performance in the first set was significantly increased with CHOCAF condition compared to PLA in both males (*p* = 0.01) and females (*p* = 0.02). Bench press endurance was similar for all conditions in both genders (*p* > 0.05). Cognitive performance was significantly increased with CHOCAF compared to PLA in males (*p* = 0.03) and females (*p* = 0.02).

**Conclusion:**

Combined CHO and CAF mouth rinsing significantly improved lower body muscular endurance and cognitive performance in both males and females.

## Introduction

Carbohydrate (CHO) and caffeine (CAF) ingestion are supported by strong evidence to have beneficial effects on exercise and cognitive performance and used by professional and/or highly trained athletes to increase training and match performance [1–3]. CAF binds to adenosine receptors A_1_ and A_2_, reducing the influence of the parasympathetic system and increasing the synthesis of neurotransmitters such as dopamine and catecholamines, increasing cognitive performance [4], tension, vigor and perception of vitality and a reduction between training load and ratings of perceived exertion (RPE) on elite and moderately trained athletes [5]. At the peripheral level, CAF improves sodium-potassium (Na^+^-K^+^) pump activity and increases the bioavailability of calcium (Ca^2+^) in the myoplasm [6]; mechanisms that explain an enhancement of one repetition maximum (1-RM) [7] power output with a determined load [8] and muscular endurance [9] in resistance exercises. A line of emerging enquiry, “mouth rinsing” without swallowing, developed two decades ago, suggests that CHO and/or CAF mouth rinsing may increase aerobic endurance-sprint type activities and cognitive performance via central mechanisms [10–12]. CAF and CHO mouth rinses are alleged to stimulate adenosine, bitter and sweet taste receptors found in the mouth, respectively, and in turn enhance activation of the sympathetic nervous system and increase brain activity related to reward and motor control [13, 14]. However, in a recent systematic review, Ehlert et al. [15] concluded that of 11 studies, only three studies found improvements of CAF mouth rinsing on exercise performance or only suggestive benefits. Moreover, Doering et al. [16] reported that 35 mg of CAF mouth rinsing for 10 s did not significantly enhance cycling time-trial performance. Therefore, further study is warranted to determine whether CHO added to CAF exerts additional ergogenic benefits [16].

Furthermore, whether CHO and CAF rinsing impacts strength and muscular endurance performance is less well known. CHO and CAF mouth rinse has potential to activate the prefrontal cortex (orbitofrontal and dorsolateral), regions associated with cognition, attention and reward, which could exert a central role in the motor control process and subsequently increase resistance exercise performance [11, 15]. To the best of our knowledge, only Clarke et al. [17] used CAF rinsing in a study design that investigated effects on muscle performance; they concluded that rinsing with a 1.2% of CAF solution either independently or combined with 6% of CHO has no significant effect on maximum strength or muscular endurance performance. Clarke et al. [17] suggested that increased dosage and/or increased number of rinses may be required to produce an ergogenic effect. Moreover, Painelli et al. [18] first demonstrated that 6.4% CHO rinsing did not improve bench press 1-RM or endurance performance (70% of 1-RM) in strength-trained athletes. The lack of an ergogenic effect was attributed to the training status of participants because resistance-trained individuals present little to no neural activation deficits in upper-body exercises [18]. Further research has been suggested [18] as decreasing exercise intensity to more muscular endurance-oriented activity is required to detect subtle benefits of mouth rinsing. Nevertheless, Bastos-Silva et al. [19] reported CHO rinse increased repetitions of bench press exercise at 80% of 1-RM in males by standardizing the duration of concentric and eccentric phases of the movement. Separate mouth rinsing studies reported diverse ergogenic outcomes due to the variable test procedures, such as dose and duration of rinse, exercise selection, prandial/training status and gender [11, 15].

Neuronal recovery following repetitive dynamic muscular contractions is vital to performance maintenance especially during resistance exercise training. Although relatively little is known about the acute neural responses to resistance exercise between sex, females were reported to be more resistant to fatigue and quicker to recover from fatiguing exercise than males in tasks utilizing low intensity loads and a slow repetition velocity in both concentric and eccentric phases [20]. Recently, sex-specific modulation of the corticospinal pathway with disparate mechanisms of cortico-motor regulation was observed, despite the same magnitude of neuromuscular fatigue in response to resistance training [21]. Due to CAF and CHO mouth rinsing known to improve muscle performance via supraspinal mechanism involving the central nervous system [11, 15], the erogenicity of mouth rinsing may vary between genders during resistance exercise training. Recently, 6.4% CHO mouth rinse in 18 male and 18 female participants did not show any sex-specific differences during bench press repetitions to failure at 65% of 1-RM [22]. Although males and females experience similar benefits from CAF ingestion [23], future research was suggested by Ehlert et al. [15] in their systematic review, to determine whether sex-specific differences exist for the mouth rinsing protocol. Additionally, bitter taste perception, which has been suggested to be the main mechanism for CAF mouth rinsing [15], may also be modified by smoking but this aspect has not been assessed or reported by previous research.

CHO and CAF mouth rinsing have been reported to reduce mental fatigue [14], enhance information processing in terms of both speed and accuracy [12], and increase reaction time due to the subsequent activation of both the orbitofrontal and dorsolateral prefrontal cortexes [13]. However, none of the above studies examined the combined effect of CHO and CAF rinse on resistance exercise and cognitive performance by directly comparing both sexes. Therefore, the aim of this study was to investigate the combined and separate effects of CHO and CAF mouth rinsing on strength, muscular endurance and cognitive performance in both male and female athletes.

## Methods

### Participants

Fourteen female and thirteen male healthy, resistance-trained team sport athletes volunteered to participate in this study (Table 1). All participants had previous experience of at least 3 times per week of resistance training for the previous 1 year, including full squat and bench press exercises in their training routine. All participants declared that they did not use creatine, steroids or oral contraceptives. Daily CAF intake levels were measured with an adapted version of the CAF intake questionnaire [24]. To abstain heterogeneity in daily intake levels of participants, only CAF naïve individuals were included in this research. All participants were very light CAF consumers (< 25 mg/day), free from musculoskeletal disorders and non-smokers to exclude the purported moderating effect of smoking on bitter taste perception [15]. Written informed consent was obtained from all participants, and the study procedures followed the principles outlined in the Declaration of Helsinki, and were approved by the Non-interventional Clinical Research Ethics Committee from Ankara University (13–816-17).

**Table 1:** Characteristics of the participants

	Age (year)	Height (cm)	Weight (kg)	Training History (year)	Caffeine Intake (mg/day)
**Female (*n*** **= 14)**	21 ± 1	170 ± 5	68 ± 6	3 ± 1	21 ± 2
**Male (*n*** **= 13)**	24 ± 3	184 ± 7	84 ± 8	5 ± 1	20 ± 3

### Experimental design

**Fig. 1:**
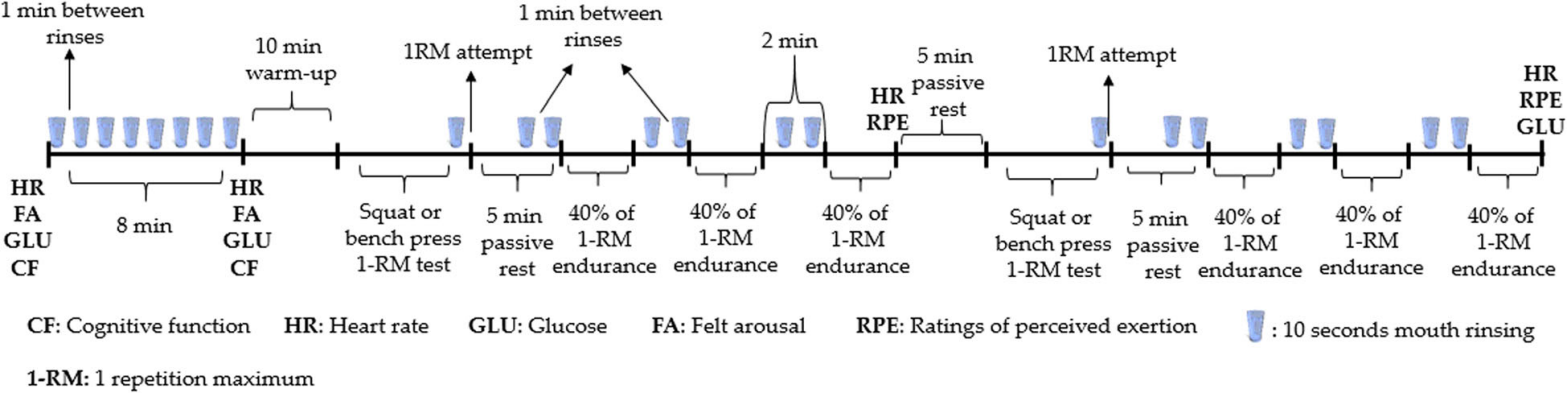
A schematic representation of the experimental protocol

A double-blind, randomised, counter-balanced and crossover research design was used. Each participant attended the laboratory on 6 occasions separated by 48–96 h to allow recovery. The first 2 sessions were familiarization and the following 4 sessions were employed to complete the test protocol with the mouth rinsing of 6% carbohydrate (CHO), 2% caffeine (CAF), combined carbohydrate and caffeine (CHOCAF) and water as a placebo (PLA). Previously, doses of 6% CHO and 2% CAF mouth rinsing were found to be beneficial on exercise performance [11, 15]. During the initial 2 familiarization sessions, all testing procedures were practiced using plain water as a mouth rinsing protocol. Squat and bench press 1-RM strength tests were conducted according to Richardson and Clarke [17]. Muscular endurance performance was tested with 40% of 1-RM repetitions to failure. Participants were introduced to the felt arousal scale (FAS) [25] to monitor arousal before and after performance of the cognitive test protocol. Upon arrival at the testing site, participants’ heart rate (HR), capillary glucose (GLU) and FAS ratings were measured, followed by cognitive function (CF) measurements followed by 8-min passive rest during which serial mouth rinses once a min (8 times in total) were performed before testing CF once again. Participants then warmed up for 10-min on a treadmill followed by 1-RM and 3 sets of 40% of 1-RM repetitions to failure test with 2-min rest between sets for squat and bench press (5-min rest between squat and bench press exercises). HR (Polar Team 2 telemetric system, Finland), ratings of perceived exertion (RPE), (GLU) (Accutrend Plus, Roche Diagnostics, Mannheim, Germany) from a fingertip and FAS were measured at different time points throughout the test protocol (see Fig. 1 for more information). Participants took part in all sessions in the morning (7:00–8:00 h) following 12 h’ overnight fasting. Participants were asked to avoid CAF and alcohol intake and physical exercise in the 12 h leading up to each visit. Furthermore, participants recorded 24-h dietary intake before the first test session and were asked to replicate the same diet prior to each main trial to standardize macronutrient consumption. Participants were also asked before each testing session to adhere to diet and lifestyle procedures. The test procedures are summarized in Fig. 1.

### Strength (1-RM) and muscular endurance (40% of 1-RM) test protocols

Following 10 repetitions with light resistance weights (20 kg), participants rested for 1-min followed by a further 3–5 repetitions with 10 and 20% added resistance for barbell bench press and back full squat, respectively. After a 2-min passive rest, participants performed 2–3 repetitions with a near-maximum resistance. The first 1-RM attempt was performed after the resistance was increased by 5–10% for bench press and 10–20% for squat exercises following 3-min passive rest. If participants lifted successfully, following the 3-min passive rest, the resistance was increased again by 10–20% for squat and 5–10% for bench press and a further 1-RM was attempted. If unsuccessful, the resistance was reduced by 2.5–5% for bench press and 5–10% for squat exercises for another 1-RM attempt after a 3-min passive rest. Strength performance (1-RM) was measured in 3–5 steps as previously described [2, 17, 19, 26]. After 1-RM was determined, and following 2 min passive rest, the resistance was reduced to 40% of 1-RM; thereafter, participants performed 3 sets of repetitions to failure with 40% of 1-RM with 2 min passive rest between sets for the squat and bench press. Squat and bench press exercises were performed on a Smith machine (Esjim, Eskişehir, Turkey) and on a rack with safety bars and Olympic plates (Esjim, Eskişehir, Turkey), respectively. To standardize technique, a certified personal trainer checked the participants and provided feedback as appropriate. Repetition tempo during muscular endurance test was standardized to 2 s for both eccentric and concentric phases using a metronome [19]. Repetition process during squat and bench press was standardized according to previous studies [2, 17]. Bar grip and foot positions were standardized for each participant and this was replicated for the subsequent testing sessions. Muscular endurance performance was recorded with total repetition number [17, 19, 22]. Being unable to maintain proper technique and posture, big oscillations in the movement speed over three consecutive repetitions and voluntarily termination were determined as criteria to end the muscular endurance test.

### Mouth rinsing protocol

During each test session, participants were given a 25-ml bolus of either 6% (1.5 g) maltodextrin (CHO), 2% (500 mg) caffeine (CAF), combined maltodextrin and caffeine (CHOCAF), or water (PLA). Solutions were rinsed around the buccal cavity for 10 s and then expectorated into a plastic cup. Each solution was administered 8 times once a min during 8 min prior to second CF test and immediately before each attempt in the 1-RM test and at each min (2 times in total) between sets in the muscular endurance test. All solutions were flavoured with 300 mg of sucralose and were similar in appearance. The same investigator prepared the solutions using electronic laboratory scales and distilled water at room temperature.

### Cognitive function

A modified arrow flanker task was performed using appropriate software (InquisitLab 5.0, Milliseconds) [27, 28] to measure cognitive function. A central yellow fixation star was presented in the center of the screen, which was replaced by five arrowheads to be responded by participants indicating the direction of center arrowhead. Arrows were presented for 200 ms on a white background with a response window of 1000 ms. There were four equiprobable conditions, two congruent (< < < < < or > > > > >) and two incongruent (< < > < < or > > < > >), and participants were asked to respond as quickly and accurately as possible to the direction of the middle target arrow by pressing corresponding response buttons. Participants performed cognitive function measurements, lasting approximately 3 min, by wearing earplugs, kept in the same body posture and in random trial order. Mean response accuracy (%) and response times (ms) were used as an index of cognitive performance [28].

### Statistical analysis

All data was analyzed using the IBM SPSS statistics for Windows (version 22.0; IBM Corp., Armonk, NY, USA). Data were analyzed using three or two-way analysis of variance (ANOVA) for repeated measures to examine main effects for 1) condition, 2) time or set and 3) condition x gender x time or set interaction. Sphericity was anaylzed by Mauchly’s test of sphericity followed by the Greenhouse-Geisser adjustment where required. If any main effect or interaction was identified, post hoc t-tests with Bonferroni adjustment was performed. Statistical significance was set at *P* < 0.05 and data is presented as mean ± SD. Intraclass correlation coefficients (ICC) were computed to assess the consistency of the four trials with conditions. The effect sizes were calculated using partial eta squared (η^2^), defined as trivial (<.10), moderate (.25–.39) or large (≥.40) [29].

## Results

### Strength (1-RM) performance

**Fig. 2:**
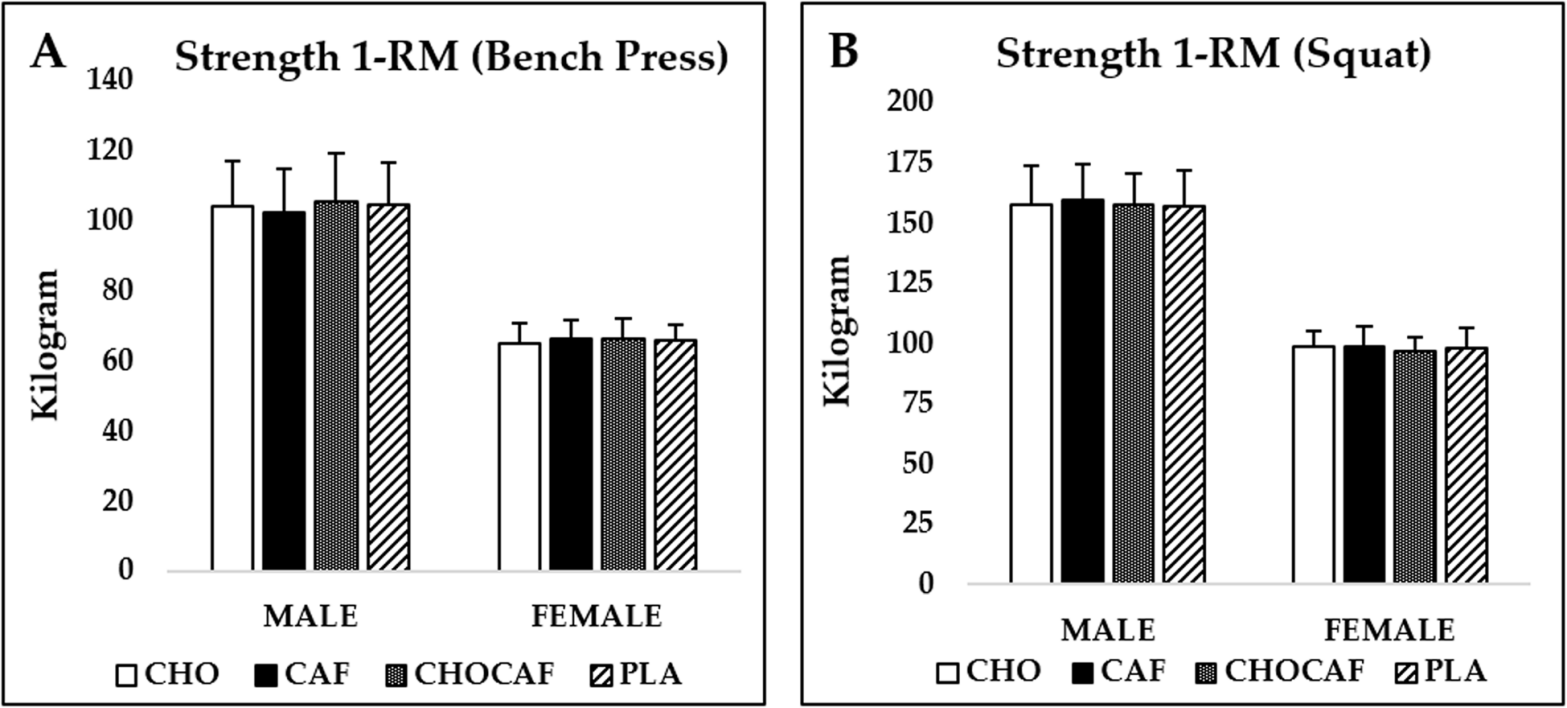
Mean ± SD bench press (**A**) and squat (**B**) 1-RM performance for males and females. CHO: carbohydrate mouth rinsing; CAF: caffeine mouth rinsing; CHOCAF: carbohydrate and caffeine mouth rinsing; PLA: placebo mouth rinsing

Condition x gender interaction was not detected either for bench press (*p* = 0.55, η^2^ = 0.14) or squat (*p* = 0.87, η^2^ = 0.02) 1-RM performance. There was no significant main effect for condition in bench press (*p* = 0.24, η^2^ = 0.12) and squat (*p* = 0.84, η^2^ = 0.01) 1-RM. As expected, males lifted significantly higher than females in bench press (*p* = 0.01, η^2^ = 0.79) and squat (*p* = 0.01, η^2^ = 0.39) (Fig. 2 A and B).

### Muscular endurance (40% of 1-RM) performance

**Fig. 3:**
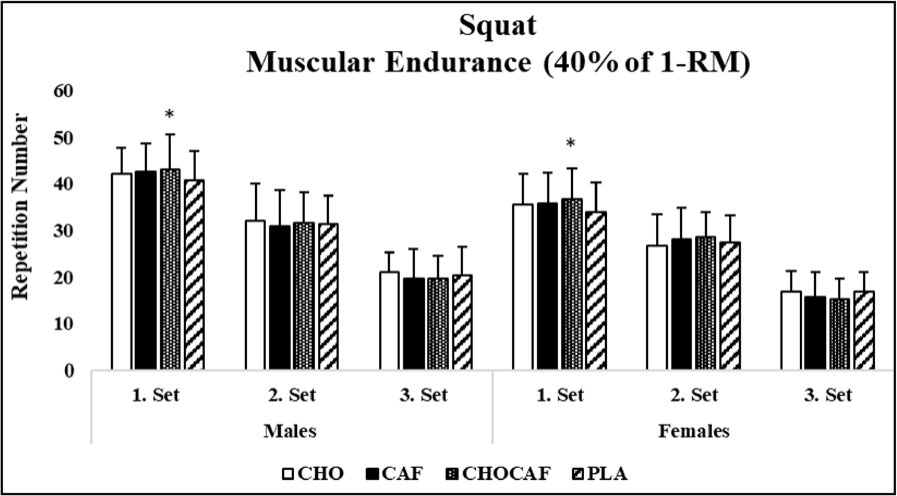
Mean (SD) repetition numbers for squat over the three sets. CHO: carbohydrate mouth rinsing; CAF: caffeine mouth rinsing; CHOCAF: carbohydrate and caffeine mouth rinsing; PLA: placebo mouth rinsing; * significantly different from PLA

**Fig. 4:**
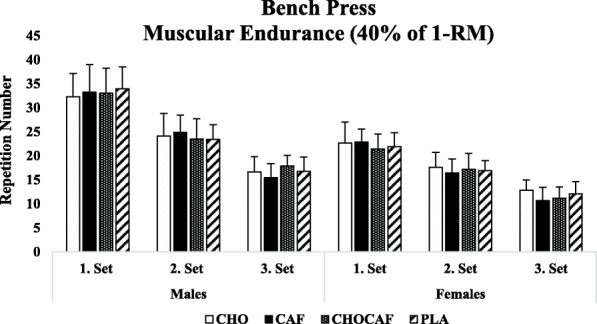
Mean (SD) repetition numbers for bench press exercises over the three sets. CHO: carbohydrate mouth rinsing; CAF: caffeine mouth rinsing; CHOCAF: carbohydrate and caffeine mouth rinsing; PLA: placebo mouth rinsing

Condition x gender x set interaction was not detected for squat (*p* = 0.29, η^2^ = 0.11) and bench press (*p* = 0.74, η^2^ = 0.15) muscular endurance performance. Gender x set (*p* = 0.82, η^2^ = 0.01; *p* = 0.72, η^2^ = 0.04) and gender x condition interaction was not significant (*p* = 0.12, η^2^ = 0.27; *p* = 0.29, η^2^ = 0.18) in squat and bench press, respectively. There was a main effect for set and gender for squat (*p* = 0.01, η^2^ = 0.97; *p* = 0.01, η^2^ = 0.73) and bench press (*p* = 0.01, η^2^ = 0.88 *p* = 0.01, η^2^ = 0.64), respectively; as expected, number of repetitions decreased from first to third set and males performed more repetitions than females in both squat and bench press muscular endurance test. Condition x set interaction was significant for squat (*p* = 0.03, η^2^ = 0.48) but not for bench press (*p* = 0.39, η^2^ = 0.20). Post-hoc analysis revealed that significantly more repetitions were performed in CHOCAF condition compared to PLA in the first set (*p* = 0.02) but there was no difference between CHOCAF and CAF (*p* = 0.33) or CHOCAF and CHO (*p* = 0.40). Further, no significant difference was detected at the second and third sets among conditions (*p* > 0.05) (Figs. 3 and 4).

ICC for squat 1-RM were 0.97 and 0.95, for muscular endurance, 0.92 and 0.94 in the first set; 0.90 and 0.91 in the second set; 0.87 and 0.90 in the third set in males and females, respectively. ICC results from bench press 1-RM were 0.98 and 0.96, for muscular endurance 0.93 and 0.90 in the first set; 0.88 and 0.93 in the second set; 0.87 and 0.90 in the third set in males and females, respectively.

### Cognitive performance

Results from the flanker task for response accuracy showed no significant condition x gender x time interaction (*p* = 0.89, η^2^ = 0.01) or main effects for gender (*p* = 0.55, η^2^ = 0.14), condition (*p* = 0.19, η^2^ = 0.17) and time (*p* = 0.22, η^2^ = 0.03) for the congruent test. Response accuracy in the incongruent test also showed no significant condition x gender x time interaction (*p* = 0.81, η^2^ = 0.01) or main effects for gender (*p* = 0.33, η^2^ = 0.04), condition (*p* = 0.13, η^2^ = 0.10) and time (*p* = 0.68, η^2^ = 0.02).

With regards to reaction time in the congruent test, there was no significant condition x gender x time interaction (*p* = 0.31, η^2^ = 0.08) or main effects for condition (*p* = 0.39, η^2^ = 0.15) and time (*p* = 0.21, η^2^ = 0.12). However, there was a main effect for gender (*p* = 0.04, η^2^ = 0.43) with males reacting faster than females in pre-rinse and post-rinse time points regardless of mouth rinsing. Although there was no condition x gender x time interaction (*p* = 0.79, η^2^ = 0.03) for incongruent test, the reaction time showed a significant condition x time interaction (*p* = 0.03, η^2^ = 0.45). Post-hoc analysis revealed that significantly faster reaction time was performed in CHOCAF (*p* = 0.02) condition compared to PLA in the post-rinse, whereas there was no difference between CHOCAF and CAF (*p* = 0.28) or CHOCAF and CHO (*p* = 0.32), respectively. Similarly, there was also a main effect of gender (*p* = 0.01, η^2^ = 0.69) for the reaction time assessed at the incongruent test, with males performing better than females in the pre- and post-rinse time in all mouth rinsing conditions. Lastly, there was no significant main effect for time (*p* = 0.78, η^2^ = 0.14) and condition for reaction time in the incongruent test (*p* = 0.63, η^2^ = 0.18) (Table 2). ICC values for cognitive performance parameters ranged between 0.91 to 0.97.

**Table 2:** Cognitive Performance

	Male				Female			
	Pre Rinse		Post Rinse		Pre Rinse		Post Rinse	
	M	SD	M	SD	M	SD	M	SD
**Response Accuracy [%] - Congruent Task**								
**CHO**	96.41	1.5	96.17	1.8	96.50	1.9	96.10	2.1
**CAF**	95.60	1.8	96.78	2.3	96.37	1.2	95.49	1.9
**CHOCAF**	95.83	1.4	95.08	1.8	95.43	2.4	95.28	2.6
**PLA**	96.86	1.9	96.72	2.4	95.90	1.5	96.13	2.2
**Response Accuracy [%] - Incongruent Task**								
**CHO**	93.98	2.9	92.46	3.1	94.06	2.7	94.93	3.1
**CAF**	92.49	2.4	93.20	2.3	94.52	3.3	94.82	2.7
**CHOCAF**	92.94	1.9	93.68	2.0	93.82	2.5	93.95	2.8
**PLA**	93.82	1.8	93.54	2.6	94.63	2.4	93.99	2.1
**Reaction Time [ms] - Congruent Task**								
**CHO**	472.45	42.9	479.26	52.9	513.21	49.2	528.49	63.1
**CAF**	460.08	54.1	465.95	42.0	524.31	30.5	512.93	39.8
**CHOCAF**	470.58	44.7	458.30	38.2	533.94	52.8	527.05	46.0
**PLA**	463.86	38.8	461.91	42.4	531.67	48.6	529.10	41.3
**Reaction Time [ms] - Incongruent Task**								
**CHO**	500.53	36.7	513.90	44.3	542.91	51.7	534.44	52.8
**CAF**	485.92	40.8	489.03	38.6	535.70	48.8	528.31	40.9
**CHOCAF**	491.55	46.3	456.08	39.4	531.20	44.1	499.30	47.5
**PLA**	504.32	55.9	509.20	51.8	538.43	43.3	536.80	41.2

*CHO* Carbohydrate mouth rinsing, *CAF* Caffeine mouth rinsing, *CHOCAF* Carbohydrate and caffeine mouth rinsing, *PLA* Placebo mouth rinsing

### Heart rate, glucose, felt arousal, RPE

Heart rate did not show significant gender x condition x time (*p* = 0.45, η^2^ = 0.04), gender x condition (*p* = 0.27, η^2^ = 0.07), gender x time (*p* = 0.12, η^2^ = 0.13) and condition x time (*p* = 0.14, η^2^ = 0.10) interaction. As expected, there was a main effect for time (*p* = 0.01, η^2^ = 0.94) with increasing HR later in exercise.

Similarly, for blood glucose there was no gender x condition x time (*p* = 0.58, η^2^ = 0.02), gender x condition (*p* = 0.19, η^2^ = 0.04), gender x time (*p* = 0.73, η^2^ = 0.02) or condition x time (*p* = 0.33, η^2^ = 0.05) interaction.

As expected RPE increased throughout exercise (main effect of time; *p* = 0.01, η^2^ = 0.79), but there was no gender x condition x time (*p* = 0.18, η^2^ = 0.07), gender x condition (*p* = 0.70, η^2^ = 0.02), gender x time (*p* = 0.31, η^2^ = 0.12) or condition x time (*p* = 0.50, η^2^ = 0.17) interaction.

Felt arousal increased over time (*p* = 0.01, η^2^ = 0.94) and there was also a condition x time interaction (*p* = 0.03, η^2^ = 0.89). Post-hoc analysis revealed significantly higher felt arousal scores after mouth rinsing in CHOCAF (*p* = 0.01) and CAF (*p* = 0.02) condition compared to PLA. However, no significant differences were observed for felt arousal in CHOCAF (*p* = 0.10) and CAF (*p* = 0.16) conditions compared to CHO. Lastly, felt arousal did not differ between gender (*p* = 0.31) and condition (*p* = 0.08) (Table 3).

**Table 3:** Heart Rate, Glucose, Felt Arousal, Ratings of Perceived Exertion

	MALE								FEMALE							
	CHO		CAF		CHOCAF		PLA		CHO		CAF		CHOCAF		PLA	
	M	SD	M	SD	M	SD	M	SD	M	SD	M	SD	M	SD	M	SD
**Heart Rate** (Beat/min)																
PreSMR	62.8	3.7	65.2	4.1	60.9	3.6	66.4	4.3	63.3	3.9	65.5	3.1	63.9	3.6	64.2	4.7
PostSMR	69.1	3.9	72.3	4.2	67.8	4.4	72.4	4.2	70.4	4.7	73.1	4.4	68.1	5.0	70.7	4.4
Middle	178.7	8.3	174.7	7.8	176.4	6.6	177.0	8.7	173.5	9.2	175.8	5.7	175.1	4.9	176.4	6.2
Posttest	184.7	10.2	186.8	9.7	180.3	8.9	186.2	9.0	184.1	8.3	180.9	6.8	181.3	7.7	180.9	7.4
**Glucose** (mg/dL)																
PreSMR	81.6	5.8	89.5	7.4	88.6	5.1	85.5	6.4	79.5	5.9	78.3	6.6	82.6	7.1	77.8	7.3
PostSMR	89.5	6.3	79.4	6.8	75.2	8.9	80.0	7.9	81.6	6.7	83.2	7.4	80.4	6.4	84.4	7.0
Posttest	80.6	5.5	81.1	6.3	83.4	5.2	80.9	6.3	79.4	5.9	82.2	6.0	82.1	7.2	80.6	6.8
**Felt Arousal** (1–6)																
PreSMR	2.1	0.7	2.4	0.8	2.1	0.6	2.2	0.9	3.0	1.0	2.8	0.9	3.1	0.3	2.9	0.8
PostSMR	3,2	1.1	3.8	1.0	3.9	1.2	2.9	1.0	4.2	1.1	4.7	0.9	4.9	1.2	4.0	1.3
**Ratings of Perceived Exertion** (6–20)																
Middle	16.4	1.7	16.0	2.0	16.3	1.8	15.9	1.4	16.9	2.0	16.4	1.5	16.8	1.8	16.0	1.6
Posttest	18.3	0.7	18.2	1.0	17.9	1.1	18.2	0.6	18.7	0.5	18.4	0.4	17.9	0.7	18.1	0.8

*CHO* Carbohydrate mouth rinsing, *CAF* Caffeine mouth rinsing, *CHOCAF* Carbohydrate and caffeine mouth rinsing, *PLA* Placebo mouth rinsing, *PreSMR* Prior to serial mouth rinsing (8 times), *PostSMR* After serial mouth rinsing (8 times), *Middle* After first muscular endurance test, *Posttest* After test protocol

## Discussion

This is the first study to directly compare the effects of CHO and/or CAF mouth rinsing on strength, muscular endurance and cognitive performance in males and females separately. Combined CHO and CAF mouth rinsing improved lower body muscular endurance and cognitive performance in both male and female athletes. However, there was no effect of CHO, CAF or CAFCHO mouth rinsing on strength and upper-body muscle performance in males or females. Lastly, serial (8 times, once per min) CAF mouth rinsing or combined with CHO increased felt arousal before cognitive function test.

Due to CHO and CAF mouth rinsing stimulating distinct brain regions associated with reward and motor control [11, 15], a combination strategy may potentiate ergogenicity on resistance exercise performance. In the current study we report for the first time a significant increase in lower body muscular endurance performance in both males and females following CHO and CAF mouth rinsing compared to PLA. These outcomes may provide indirect evidence of improved neural drive to the motor units resulting in muscle recruitment improvement [10] by which divergent mechanisms of CHO (taste receptors) and CAF (adenosine and bitter receptors) mouth rinsing [11, 13, 15]. However, a previous study [17] did not report any ergogenic effect on squat endurance, possibly due to not standardizing the repetition velocity, in turn, fluctuation in tempo may affect the result. Further, decreasing the intensity of muscular endurance test from 60% of 1-RM to 40% which is more endurance oriented, increasing dose of CAF from 1.2 to 2% and conducting in a fasted state may amplify our results compared to Clarke et al. [17]. Improvement in squat endurance in the only first set with combined but not separate rinsing of CHO and CAF partially confirmed previous study [10]. In contrast, Dolan et al. [30] found no combined effect of CHO and CAF mouth rinsing on Yo-Yo IRT-1 performance. Furthermore, author [30] mentioned that only one time 10-s rinsing dose can be well below to required stimulation of taste receptors. In the current study, although mouth rinses were provided two times in 2 min immediately before each set of muscular endurance assessments, no effect was found in second and third sets of squat and all sets of bench press endurance. Future research should investigate the optimal frequency and number of mouth rinses between resistance exercises sets.

Lower body muscular endurance performance was increased in the first set with combined (CHOCAF) but not separate CHO and CAF mouth rinsing despite the same RPE intensity. Decimoni et al. [31] demonstrated that CHO mouth rinsing lowered RPE compared to placebo during three sets of five resistance exercise to volitional fatigue in females, despite no difference between trials when examining workload of each exercise. Further, 6 g of CHO mouth rinsing was shown to improve total repetitions for both upper and lower limbs resistance exercise in females with no effect on RPE [32]. Methodological differences between the current study and these other studies – including movement tempo, number of exercise tests, duration of muscular endurance test, dose of mouth rinse, resistance intensity and training status of participants – may explain the disparity in performance and RPE data. Repetition cadence was 2 s for each concentric and eccentric contraction meaning 4 s for each repetition in the current study, but it was half of this (2 s each repetition) in the Decimoni et al. [31] study. Duration of muscular endurance tests was also shorter than the Decimoni et al. [31] study (approximately 20 min vs. 50 min). It seems positive effect of CHO mouth rinsing occurs in the more fatigued state when the number/duration of exercise, in turn, volume of training session is more than current and previous studies [17, 22, 26, 33, 34] because mere presence of CHO in the mouth may attenuate declines in motor function associated with fatigue by activating novel signaling pathway [11, 13, 14]. Furthermore, 6.4% CHO mouth rinsing was shown to significantly improve number of repetitions to volitional fatigue with a load equal to 80% of 1-RM in bench press but not in leg press exercise with 4 s cadence for each repetition [19]. There have also been reports that CHO mouth rinsing can significantly increase neuromuscular performance during an isokinetic fatiguing task [35] and sprint power output [36] with similar or higher HR and RPE values. To make firm inferences regarding effect of CHO mouth rinsing on resistance exercise performance, future research is needed with experimental designs by manipulating the duration of test protocol, repetition cadence (2 vs. 4) and resistance intensity (40% vs 80% of 1-RM) as a confounding factor.

We attempted to standardize habitual CAF intake of participants by recruiting very low daily CAF users to detect subtle benefits of mouth rinsing. Because CAF habituation may increase the number of adenosine receptors found at the mouth and thus down-regulate the sensitivity of CAF [1, 15], this setting may work on the current results that was found significant increase in lower body muscular endurance and cognitive performance with combined mouth rinsing in the early morning. Although 6 mg/kg of CAF intake was ergogenic, mouth rinsing was reported to have no influence on 3-km cycling performance in recreationally trained males with the range of daily CAF intake was 0–380 mg/day potential to including low, moderate and high habitual CAF users [37]. However, magnitude-based inferences reported that mouth rinsing of CAF “likely” enhanced performance early as opposed to late in the day partially supporting our early morning results. It is worth investigating whether CAF mouth rinsing increases exercise performance in the afternoon or evening due to some athletes preferring mouth rinsing instead of ingestion because of possible impairment of sleep quality. Further, being a low habitual CAF user was shown to impact sensitivity to CAF mouth rinsing [10]. One may speculate that habituation reduces the responsiveness to mouth rinsing. No research to date has investigated the moderating role of daily CAF intake levels of participants on CAF mouth rinses’ erogenicity by directly comparing high and low CAF users.

The current study employed a cognitive function protocol before physical tests to detect only mouth rinsing effects. Based on previous reports [14, 36] that suggested 8 times serial mouth rinsing with CHO and/or CAF can sufficiently stimulate receptors found in the oral cavity, our study for the first time has shown that combined CHO and CAF mouth rinsing once a min during 8 min (which is typical of physical warm-up time) significantly increases cognitive performance in both males and females in the very early morning and fasted state. Mechanisms responsible for the cognitive improvement may likely be the restoration of dopaminergic transmission in the striatum and anterior cingulate cortex that initiate a signal transduction cascade towards the brain by stimulating bitter and sweet taste receptors in the mouth [14]. During many team sports, such as football, basketball, handball and rugby, performing physical and cognitive tasks simultaneously and efficiently is required to win the match or beat the opponent, so mouth rinsing of CHO and CAF can also be used to increase not only physical but cognitive performance as the current research demonstrated. A few studies were conducted on this topic showed mix results. CHO rinsing attenuated the decline in executive function induced by sustained moderately high-intensity exercise [38]. However, no differences were reported between CHO and PLA rinsing on cognitive performance despite significant improvement in skill-specific fencing performance [39]. Although the current study did not show benefits of separate mouth rinsing, Pomportes et al. [12] demonstrated that CAF or CHO mouth rinsing solely can increase cognitive control and temporal performance during a submaximal exercise. Further, sole presence of CAF in the mouth without ingestion was shown to exert a likely beneficial effect on reaction time in a task requiring executive control [13]. As cognitive function with separate rinses in the current study remains to be fully elucidated, future studies, using other testing protocols (e.g., electroencephalography during physical exercise) or increasing the dose and frequency of mouth rinsing may be required.

On a practical level, it can be suggested, based on the current study’s results, that CAF-naïve male and female athletes may benefit from combined 6% CHO and 2% CAF mouth rinsing during warm-up in the very early morning. Some athletes may undergo gastro-intestinal distress, have metabolic diseases such as diabetes/celiac, may be genetically disadvantaged with alterations in CAF metabolism or may need to limit or periodize energy intake; these athletes may benefit from combined CHO and CAF mouth rinsing strategy to increase arousal, physical and cognitive performance by simply avoiding hepatic clearance with such a strategy. Further, in some instances, athletes may refuse to eat before early morning training, then current study’s outcomes can be utilized.

There are a few limitations in our study. We did not consider menstrual cycle for female athletes; however, several studies [40, 41] show no effect of menstrual cycle on sports performance. The effectiveness of blinding was not tested by asking participants to identify the solutions they had rinsed. It is not known whether expectancy effect can somehow affect the results of the current study. Although participants were instructed to replicate their 24-h diet prior to each test session, macronutrient intake was not empirically measured. Further, the inability to observe brain activity with electroencephalography and measure neurotransmitter concentrations make it difficult to fully elucidate exact mechanisms why separate mouth rinsing of CHO and CAF did not improve physical and cognitive performance but the combination of both (CHOCAF) did. Also, arousal was significantly increased with CAF solely rinsing mechanisms by which is not known due to the aforementioned reasons. Further, testing sessions were conducted in a fasted state, given that resistance training is not commonly performed in a fasted state. Lastly, by taking into consideration that capillary glucose was not different between conditions throughout test protocol, plasma CAF concentration was not measured so it is not known whether improvements in combined trial is a result of absorption of CAF in the buccal mucosa.

## Conclusions

Mouth rinsing with a 6% CHO and 2% CAF dose increased lower body muscular endurance and cognitive performance in both male and female athletes. In addition to combined rinsing, separate CAF mouth rinsing also increased arousal levels. However, these findings need to be treated with caution as training status and habituation to CAF of athletes may decrease sensitivity to mouth rinsing. In the future, chronic use of CHO and especially CAF mouth rinsing during resistance exercise should be examined to confirm these acute outcomes.

## Data Availability

Data and publication materials are available from the corresponding author on reasonable request.

## Abbreviations

CHO: Carbohydrate
CAF: Caffeine
1-RM: 1 repetition maximum
CHOCAF: CHO and CAF
PLA: Placebo
HR: Heart rate
FA: Felt arousal
RPE: Ratings of perceived exertion
GLU: Glucose
CF: Cognitive function
ICC: Intraclass correlation coefficients
